# Federated target trial emulation for time-to-event outcomes via POLARIS: Pooled-equivalent One-shot Likelihood Aggregation for Real-world Inference in Survival

**DOI:** 10.21203/rs.3.rs-9613167/v1

**Published:** 2026-05-20

**Authors:** Tingyin Wang, Yuqing Lei, Lu Li, Yudong Wang, Huilin Tang, Yue Wu, Siqi Chen, Bingyu Zhang, Jie Hu, Huiyuan Wang, Jingna Feng, Zifang Kong, Chengrong Wang, Yujia Zhou, Anastassia Amaro, Jingmei Qiu, Yuan Lu, Mei Liu, Yulun Liu, Cui Tao, Hua Xu, Yiwen Lu, Yong Chen

**Affiliations:** University of Pennsylvania; University of Pennsylvania; University of Pennsylvania; University of Pennsylvania; University of Pennsylvania; University of Pennsylvania; University of Pennsylvania; University of Pennsylvania; University of Pennsylvania; University of Pennsylvania; University of Pennsylvania; Milwaukee School of Engineering; University of Florida; Yale University; University of Pennsylvania; Milwaukee School of Engineering; Yale University; University of Florida; University of Texas Southwestern Medical Center; Mayo Clinic; Yale University; University of Pennsylvania; University of Pennsylvania

## Abstract

Heterogeneous treatment effects (HTE) are key to precision medicine, but most real-world studies lack the scale and diversity needed to detect them. While multi-site analyses offer a potential solution, data-sharing constraints often prevent access to patient-level information across institutions. We introduce POLARIS, a federated framework for time-to-event target trial emulation. POLARIS converts each site’s weighted Cox risk function into a compact tensor shared once with the coordinating center, enabling lossless reproduction of pooled estimates without sharing patient-level data. We applied POLARIS across five U.S. health systems to study risk of gastrointestinal outcomes after GLP-1 receptor agonist (GLP-1RAs) initiation versus sodium-glucose cotransporter 2 inhibitors (SGLT2is) and dipeptidyl peptidase 4 inhibitors (DPP4is). Results showed that GLP-1RAs were consistently associated with higher risks of nausea and vomiting, particularly among men, individuals with higher baseline HbA1c (≥ 8.5%), and lipid therapy. POLARIS provides a scalable solution for distributed target trial emulation and fine-grained assessment of HTE across diverse health systems.

## Introduction

Glucagon-like peptide-1 receptor agonists (GLP-1RAs) are now among the most widely used therapies for type 2 diabetes and obesity, supported by strong evidence of glycemic and cardiometabolic benefit^1^. Their use has therefore expanded rapidly, including off-label adoption among individuals without diabetes. Yet gastrointestinal (GI) adverse effects such as nausea, vomiting, and abdominal discomfort remain common and often limit tolerability and adherence. Randomized trials and large observational studies have consistently demonstrated an overall increased risk of gastrointestinal events associated with GLP-1RAs compared with placebo and active comparators, establishing the average treatment effect of these agents on GI outcomes^2–4^. However, investigations into the magnitude and clinical relevance of these risks across patient subgroups are often limited to specific, isolated subgroups^5^. Importantly, emerging data suggest that the risk and severity of such adverse effects vary across patient subgroups and drug classes, underscoring the need for systematic evaluation of heterogeneity of treatment effects (HTE) of GLP-1RAs across a broad spectrum of patient subgroups^6–8^.

Target trial emulation (TTE) has emerged as a systematic approach for applying the design principles of randomized trials to real-world data^9^. This framework supports systematic evaluation of treatment effects and subgroup heterogeneity. Reliable estimation of subgroup-specific effects requires large and diverse populations, often involving collaboration between multiple health systems. However, data-security regulations and governance frameworks (e.g., HIPAA, GDPR) often restrict the exchange of patient-level data, creating major barriers to collaborative target trial emulation^10,11^. Federated learning offers a potential solution by enabling distributed analyses without data transfer, but current methods typically trade-off between statistical fidelity and communication efficiency, and few can achieve both.

In response, we developed **POLARIS** (Pooled-equivalent One-shot Likelihood Aggregation for Real-world Inference in Survival), a federated framework for time-to-event target trial emulation. It applies explicit protocols and uses inverse probability of treatment weighting (IPTW) to estimate treatment effect. POLARIS transforms each site’s data into a storage-efficient tensor representation using tensor trains (TT). These tensors are then aggregated to recover the pooled Cox hazard estimator. To our knowledge, this is the first federated framework for TTE of time-to-event outcomes that achieves both statistical fidelity and communication efficiency. It recovers pooled estimates exactly (lossless) using only a single round of summary statistics transfer (one-shot), without sharing patient-level data.

We demonstrate the utility of POLARIS across five large-scale health systems for high-throughput screening of HTEs. The five systems include the University of Pennsylvania Health System (UPHS), Yale New Haven Health System (YNHHS), Mayo Clinic, OneFlorida+ Clinical Research Network, and UT Southwestern Medical Center (UTSW). We evaluate subgroup-specific risk of GI outcomes associated with GLP-1RAs compared to sodium-glucose cotransporter 2 inhibitors (SGLT2is) and dipeptidyl peptidase 4 inhibitors (DPP4is). This use case illustrates how POLARIS can be applied in practice to support multi-institutional TTE, particularly in settings where data cannot be pooled but consistent analytic protocols can be maintained.

## Results

### Overview

POLARIS performs time-to-event target trial emulation in distributed settings by combining IPTW with tensor-based aggregation. It reproduces estimates identical to pooled analyses using only a single round of summary-level communication, without sharing patient-level data.

**Figure 1:**
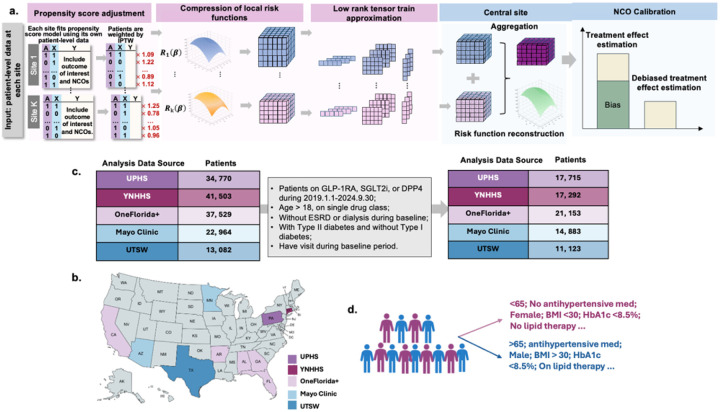
Overview of the POLARIS framework and study design. (a) Federated analytic workflow: local IPTW and weighted Cox models compressed into tensor-train summaries and aggregated once at the POLARIS hub to yield lossless estimates without sharing individual-level data. (b) Maps of five participating health systems. (c) Five participating health systems federated data overview. (d) Subgroups/interaction terms we consider.

The overall framework is illustrated in Fig. 1a. It first reconstructs the pooled inverse probability weighted Cox model exactly using tensor-based aggregation of summary statistics. By evaluating weighted partial likelihood components over a standardized parameter grid and compressing them into a lossless tensor representation, POLARIS enables the coordinating center to recover pooled estimates without accessing individual-level risk sets. The procedure requires only a single round of communication and preserves patient privacy while maintaining full statistical equivalence to centralized analysis.

We applied POLARIS across five large U.S. health systems to evaluate subgroup-specific risk of GI outcomes among adults initiating GLP-1RA compared to those initiating SGLT2is or DPP4is. Baseline characteristics are summarized in Table 1, and additional details of the target trial emulation framework and covariate balance are provided in Supplementary Figures S4a-1 through S4e-2. This deployment demonstrates how POLARIS enables multi-institutional analyses by aggregating summary information across sites. Its one-shot communication and lossless estimation support scalable evaluation of treatment effects across distributed health systems.

### Exact reconstruction of pooled weighted Cox inference via tensorized aggregation

In federated time-to-event TTE, the primary inferential objective is to recover the pooled weighted Cox estimating equations without sharing individual-level risk-set data. To empirically verify this reconstruction property for POLARIS, we conduct an internal validation using data from Penn Medicine. The dataset was partitioned into six sites based on hospital ID to simulate a multi-site federated setting. Each subset was analyzed locally to generate summary outputs, which were then aggregated at a coordinating center following the POLARIS workflow. Further technical details regarding the grid-based function evaluation and tensor-train compression are provided in Supplementary Section S3.

**Figure 2:**
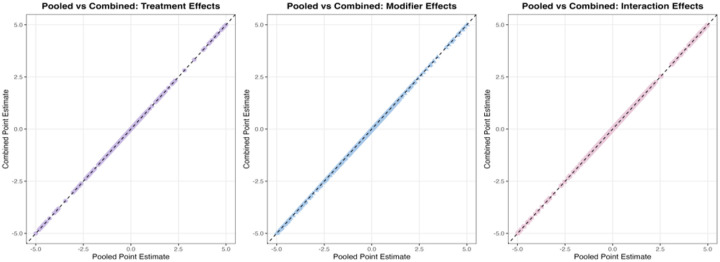
Comparison of point estimates for three Cox regression coefficients, including the treatment effects, potential modifier effects, and interaction effects, obtained using POLARIS and a centralized pooled analysis. Each point represents one coefficient, with shapes and colors indicating the different terms. The dashed line denotes the identity line (y = x). The near-perfect alignment demonstrates that POLARIS reproduces pooled estimates and standard errors within numerical precision, confirming its lossless property.

As shown in Fig. 2, POLARIS estimates were numerically indistinguishable from pooled estimates across treatment, potential effect modifier, and interaction terms, with all points falling along the identity line. This confirms that tensor aggregation exactly reconstructs the pooled risk function, thereby reproducing the same coefficients and standard errors as pooled estimation.

### POLARIS discovers HTEs in GI outcome across subgroups of GLP-1RA vs SGLT2i

After validating POLARIS using internal data from Penn Medicine, we applied the framework across five federated health systems: UPHS, YNHHS, OneFlorida+ Clinical Research Network, Mayo Clinic Health System, and UTSW. Additional details of data description are provided in the Supplementary Material Section S1, cohort construction and data preprocessing procedure are provided in the Supplementary Material Section S2 Part A and Part C. Both drug classes are recommended and widely used for type 2 diabetes but differ substantially in mechanism and GI side effects. SGLT2is therefore serves as an appropriate active comparator for isolating the gastrointestinal risks specifically associated with GLP-1RAs, while reducing confounding due to differences in treatment indication.

**Figure 3:**
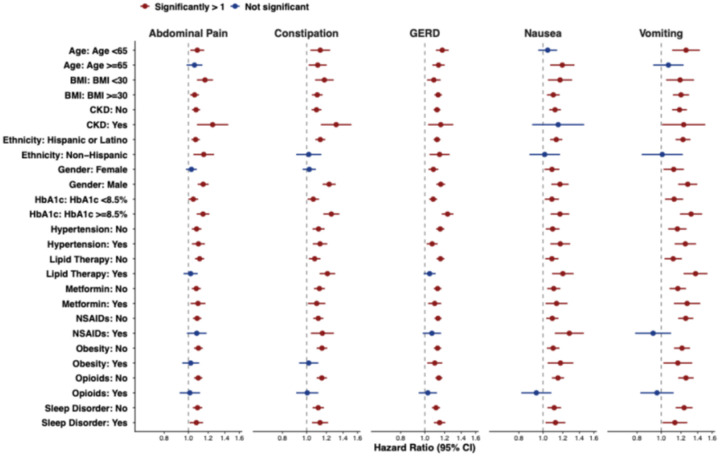
Forest plots of treatment effects of GLP-1 RAs vs SGLT2is across predefined subgroups. They display subgroup-specific hazard ratios and 95% confidence intervals for each outcome, stratified by demographic, medical, medication, laboratory, and behavioral or healthcare-related factors. Values above 1 indicate higher risk with GLP-1RAs relative to SGLT2is.

Figure 3 presents subgroup analyses evaluating heterogeneity in GI outcomes associated with GLP-1RA initiation compared with SGLT2i initiation. Complete subgroup-specific hazard ratios and 95% confidence intervals for all outcomes and effect modifiers are provided in Supplementary Data File 1. Site-specific event counts and event rates across all subgroups and outcomes are provided in Supplementary Data File 2. Across the five outcomes (abdominal pain, constipation, GERD, nausea, and vomiting), the overall treatment effect consistently indicated a higher risk of GI symptoms among GLP-1RA users. However, the magnitude of this association varied across clinically relevant patient characteristics.

Several potential effect modifiers showed consistent patterns across outcomes. Individuals with higher baseline HbA1c (≥ 8.5%) tended to experience greater increases in GI risk associated with GLP-1RA use than lower baseline HbA1c (< 8.5%), particularly for abdominal pain (HR = 1.14, 95% CI: 1.08–1.21, *p-value*: <0.001 vs 1.05, 1.00–1.09, 0.05), constipation (1.26, 1.17–1.35, < 0.001 vs 1.06, 1.01–1.12, 0.023), and GERD (1.23, 1.17–1.30, < 0.01 vs 1.08, 1.04–1.12, < 0.01). A similar pattern was observed by sex, with men generally experiencing larger relative risks than women, including for abdominal pain (1.07, 1.03–1.12, < 0.001 vs 0.99, 0.95–1.03, 0.520), constipation (1.19, 1.10–1.28, < 0.001 vs 1.02, 0.96–1.07, 0.57), GERD (1.12, 1.06–1.18, < 0.001 vs 1.04, 1.01–1.08, 0.023), and vomiting (1.25, 1.15–1.35, < 0.001 vs 1.07, 1.01–1.14, 0.021). Medication use and comorbidity also influenced risk heterogeneity. Baseline use of lipid therapy was associated with greater risks for certain outcomes, particularly constipation (1.21, 1.13–1.30, < 0.001 vs. 1.06, 1.01–1.11, 0.012) and vomiting (1.37, 1.23–1.53, < 0.001 vs 1.10, 1.04–1.17, < 0.001). However, individuals with baseline NSAID use (0.93, 0.72–1.20, 0.569 vs 1.25, 1.02–1.54, 0.033) or opioid use (0.96, 0.75–1.23, 0.760 vs 1.25, 1.02–1.54, 0.033) tended to have a lower risk of vomiting compared with those without use. Individuals with chronic kidney disease tend to have an increased risk of abdominal pain (1.25, 1.08–1.45, 0.003 vs. 1.07, 1.03–1.11, < 0.001) and constipation (1.27, 1.13–1.41, < 0.001 vs. 1.07, 1.02–1.11, 0.002), compared with those without chronic kidney disease. Together, these findings highlight potential heterogeneity in GI symptom risk across patient subgroups treated with GLP-1RAs compared to SGLT2is.

### POLARIS discovers HTEs in GI outcome across subgroups of GLP-1RAs vs DPP4is

**Figure 4:**
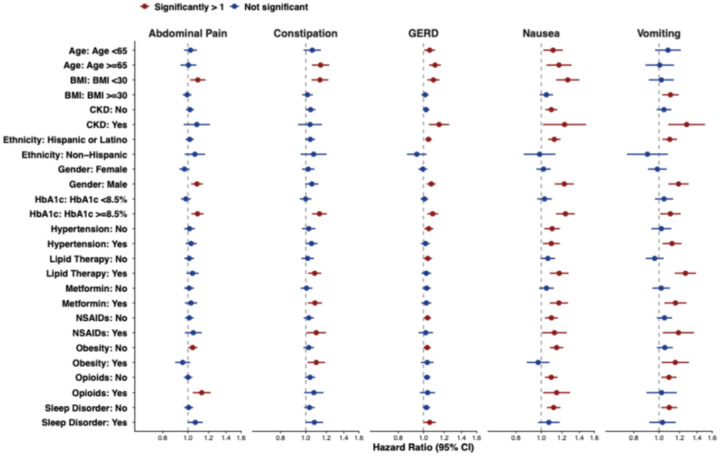
Forest plots of treatment effects of GLP-1 RAs vs DPP4is across predefined subgroups. They display subgroup-specific hazard ratios and 95% confidence intervals for each outcome, stratified by demographic, medical, medication, laboratory, and behavioral or healthcare-related factors. Values above 1 indicate higher risk with GLP-1RAs relative to DPP4is.

Figure 4 presents subgroup-specific HRs for five GI outcomes associated with GLP-1RA initiation compared with DPP4is initiation. Overall, GLP-1RA use was associated with modestly higher risks of GI symptoms, including abdominal pain, constipation, GERD, nausea, and vomiting. However, the magnitude of these associations varied across clinically relevant patient characteristics, suggesting heterogeneity in treatment effects across subgroups. Notably, a similar pattern of subgroup variation was observed in the comparison of GLP-1RAs versus SGLT2is, although differences in certain subgroups (e.g., NSAID and opioid use) were attenuated, indicating consistent heterogeneity in GI risk across different active comparator analyses.

### False discovery rate control for subgroup interaction effects

**Figure 5:**
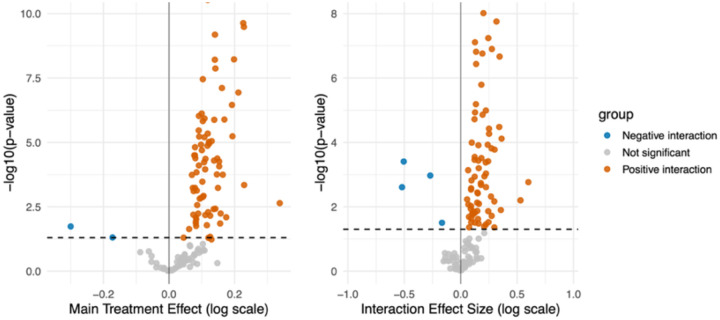
Volcano plots of main effects (left) and interaction effects (right) after Benjamini–Hochberg false discovery rate correction. The x-axis shows effect estimates on the log scale, and the y-axis shows −log10 (p-values). The dashed horizontal line indicates the significance threshold.

To account for multiple testing across outcomes and subgroup interactions, we controlled the false discovery rate using the Benjamini–Hochberg procedure (q < 0.10). Overall treatment effects of GLP-1RAs on GI outcomes remained significant after FDR correction. A subset of subgroup interaction effects also remained robust. In the GLP-1RA versus SGLT2i comparison, persistent signals were observed primarily for nausea and vomiting among patients with chronic kidney disease, elevated baseline HbA1c, and obesity. Similar patterns were seen in the comparison with DPP4is, particularly among individuals with chronic kidney disease and baseline NSAID or opioid use. Several nominal subgroup differences did not survive FDR adjustment, indicating effective control of false-positive findings. The interaction effects that remained significant were supported by large patient numbers and multi-site consistency, underscoring their reproducibility. The results were summarized and visualized using the volcano plots (see Fig. 5).

## Discussion

This study introduces POLARIS, a framework for time-to-event TTE across distributed EHR networks without sharing patient-level data. POLARIS combines propensity score weighting and tensor-based aggregation to recover centralized results using only summary-level data^12^, with no need to transfer patient-level records. This design makes POLARIS well suited for large-scale networks where data access is restricted but consistent analytic protocols can be maintained.

We applied POLARIS across five major U.S. health systems to evaluate GI risks associated with GLP-1RAs compared with SGLT2is and DPP4is. Across both active comparator analyses, GLP-1RA initiation was consistently associated with higher risks of GI symptoms, including abdominal pain, constipation, gastroesophageal reflux disease, nausea, and vomiting. Although the magnitude of these associations varied across outcomes, the largest relative increases were observed for nausea and vomiting. Importantly, POLARIS identified clinically meaningful heterogeneity in these risks across patient subgroups. Patients with higher baseline HbA1c (≥ 8.5%) tended to experience greater increases in GI risk following GLP-1RA initiation, and men generally showed larger relative risks than women. Comorbid conditions and concomitant medications also modified risk patterns, with chronic kidney disease associated with higher risks of abdominal pain and constipation, lipid-lowering therapy linked to greater risks of constipation and vomiting, while NSAID and opioid use were associated with lower risk of vomiting. Notably, similar patterns of subgroup variation were observed when comparing GLP-1RAs with both SGLT2is and DPP4is, suggesting that certain patient characteristics may predispose individuals to GLP-1RA–related GI adverse effects regardless of the comparator therapy. Together, these findings demonstrate the ability of POLARIS to detect coherent and clinically meaningful heterogeneity in treatment safety across distributed health systems and highlight its potential to support more individualized treatment decisions.

This study highlights several advantages of the POLARIS framework. By design, POLARIS delivers analytical fidelity equivalent to pooled modeling while performing all computations locally and exchanging only a single set of summary statistics, thereby maintaining local computation and minimizing cross-site communication. Applied across five health systems, the framework enables robust and reproducible estimation of heterogeneous treatment effects for GI outcomes across diverse demographic and clinical contexts. In addition, the framework facilitates rigorous causal inference by enabling consistent implementation of propensity score weighting and outcome modeling across sites, producing more reliable and interpretable effect estimates. Collectively, these features position POLARIS as a scalable framework for generating actionable and trustworthy HTE evidence from real-world data^13^.

Our study also has several potential limitations. First, exposure and outcome definitions were based on structured EHR data, which may be incomplete or inconsistently recorded across sites^14–16^. This could lead to missing information on medication use or treatment discontinuation and may result in underreporting of GI adverse events. Second, although POLARIS accounts for observable cross-site heterogeneity by allowing each health system to estimate its own propensity scores and weighted risk sets before aggregation, residual differences in unmeasured clinical practices, coding conventions, or data-capture processes may persist across health systems^17^. These system-level variations are inherent to multi-site real-world data and may introduce heterogeneity that is not fully addressed by the current covariate set. Third, the current implementation of POLARIS focuses exclusively on structured EHR elements and does not incorporate unstructured data such as clinical notes, imaging, or free-text laboratory narratives. Future extensions may enhance phenotype precision and clinical depth by integrating these data sources through federated AI techniques that jointly model structured and unstructured information while preserving data locality.

## Methods

### Study Cohort and Variable Definitions

#### Data source

The study was conducted across five major U.S. academic health systems: University of Pennsylvania Health System (UPHS, 7 hospitals serving 6.5 million patients), Yale New Haven Health System (YNHHS, 4 million patients), OneFlorida+ Clinical Research Network (OneFlorida+, 24 million patients), Mayo Clinic Health System (Mayo, 10 million patients) and UT Southwestern Medical Center (UTSW, 2 million patients). Each institution maintains comprehensive longitudinal electronic health record repositories standardized for diagnoses (ICD-9/10 codes), medications (RxNorm), and laboratory measurements (LOINC). All datasets were fully de-identified prior to analysis, and local Institutional Review Board (IRB) approval or exemption was obtained at each participating site. Detailed descriptions of data preprocessing procedures, variable harmonization, and cohort construction are available in the *Supplementary Appendix*.

### Target Trial Emulation Framework

#### Cohort Construction:

We designed and emulated two pragmatic target trials to compare gastrointestinal outcomes among adults with type 2 diabetes: (1) initiation of GLP-1RAs versus SGLT2is and (2) initiation of GLP-1RAs versus DPP4is. Eligible participants were adults aged 18 years or older who newly started one of the study medications between January 1, 2019, and September 30, 2024. We excluded individuals with any prior exposure to study drugs, a diagnosis of type 1 diabetes or end-stage renal disease and required each patient to have at least one clinical encounter during the 12-month baseline period preceding drug initiation. Among these, we identified patients who initiated either a GLP-1RAs, DPP4is, or SGLT2is during the study period. Treatment initiation was defined as the first prescription for a given drug class during the 12 -month baseline. The full target trial protocol and its operationalization in EHR data are detailed in Supplementary Appendix Table S1. Patient attrition following sequential application of eligibility criteria at each participating site is summarized in Supplementary Figures S1a–S1e.

##### Treatment

Treatment strategies were defined as initiation of GLP-1RAs, DPP4is or SGLT2is. Detailed list of agents provided in the Supplementary Appendix Table S2. The index date was defined as the date of first prescription fill for the study medication among patients without prior use of these drug classes.

##### Covariates

Baseline covariates were extracted from structured EHR data one year prior to the index date. These included age, sex, race/ethnicity, and a set of predefined comorbidities based on ICD-10 diagnosis codes, including diabetes with complications, hypertension, heart failure, chronic pulmonary disease, depression, and obesity. Medication history was identified using RxNorm ingredient codes (complete list in Supplementary Appendix Table S2), capturing baseline exposure to relevant drug classes such as metformin, insulin, sulfonylureas, and antidepressants. All covariates were converted to categorical or binary indicators as appropriate, with missing values treated as a separate category when applicable. Pre-imputation missingness rates for laboratory variables across sites are reported in Supplementary Table S3. Imputation diagnostics, including three-stage distributional comparisons and observed-versus-imputed comparisons for each laboratory variable at each site, are shown in Supplementary Figures S2a–S2e and S3a–S3e, respectively.

#### Outcome:

The primary outcomes were the time to first occurrence of GI adverse events, identified using validated ICD-9/10 diagnosis codes in the Supplementary Appendix Table S2. Specifically, we evaluated five clinically relevant GI outcomes: abdominal pain, constipation, gastroesophageal reflux disease (GERD), nausea, and vomiting. Each outcome was defined as the first outpatient or inpatient encounter coded for the corresponding condition following drug initiation.

We additionally assessed heterogeneity of treatment effects across key demographic, clinical, and laboratory subgroups, including age, sex, baseline metabolic control, and gastrointestinal history (see Supplementary Appendix Table S2 for details).

##### Defining Potential Effect Modifiers

We evaluated treatment effect heterogeneity across clinically relevant subgroups including age (< 65 vs ≥ 65 years), gender (female vs male), ethnicity (Hispanic or Latino vs Non-Hispanic), medical history (chronic kidney disease, hypertension, obesity, sleep disorder), medication history (metformin, lipid therapy, opioids, NSAIDs), and laboratory thresholds (HbA1c ≥ 8.5% vs < 8.5%, BMI ≥ 30 vs < 30 kg/m^2^). Operational definitions for each binary modifier are provided in Supplementary Table S4.

## Supplementary Material

Supplementary Files

This is a list of supplementary files associated with this preprint. Click to download.

- 1.xlsx
- 2.xlsx
- POLARISsupp.docx

## Figures and Tables

**Table 1:** Baseline characteristics are presented for each participating site (UPHS, YNHHS, Mayo Clinic, OneFlorida+, and UT Southwestern) stratified by treatment group (SGLT2is, GLP-1RAs, and DPP4is). Continuous variables are reported as mean (standard deviation), and categorical variables are presented as number (percentage). Health care utilization measures include inpatient, outpatient, and emergency department encounters in the year prior to index date.

Comparison Groups	UPHS			YNHHS			Mayo			OneFlorida+			UT Southwestern		
	SGLT2 Cohort (N = 9,713)	GLP1 Cohort (N = 8,002)	DPP4 Cohort (N = 6,741)	SGLT2 Cohort (N = 8,104)	GLP1 Cohort (N = 9,188)	DPP4 Cohort (N = 6,550)	SGLT2 Cohort (N = 8,715)	GLP1 Cohort (N = 13,518)	DPP4 Cohort (N = 2,845)	SGLT2 Cohort (N = 6,584)	GLP1 Cohort (N = 4,651)	DPP4 Cohort (N = 3,468)	SGLT2 Cohort (N = 5098)	GLP1 Cohort (N = 3,562)	DPP4 Cohort (N = 1,093)
Age at Entry mean (SD)	64.10 (11.99)	57.50 (12.76)	66.15 (13.08)	63.98 (12.89)	57.21 (13.58)	64.71 (14.40)	66.19 (12.30)	59.76 (12.62)	65.50 (12.88)	60.58 (12.49)	55.04 (12.96)	62.02 (14.00)	63.80 (12.60)	57.69 (13.55)	65.99 (12.70)
Sex															
Female	3, 503 (36.1%)	4, 357 (54.4%)	3, 155 (46.8%)	2, 941 (36.3%)	5, 175 (56.3%)	3, 015 (46.0%)	2, 862 (32.8%)	6, 557 (48.5%)	1, 147 (40.3%)	2, 691 (40.9%)	2, 667 (57.3%)	1, 689 (48.7%)	1, 163 (39.3%)	2, 016 (56.6%)	493 (47.4%)
Male	6, 210 (63.9%)	3, 645 (45.6%)	3, 586 (53.2%)	5, 163 (63.7%)	4, 013 (43.7%)	3, 535 (54.0%)	5, 853 (67.2%)	6, 961 (51.5%)	1, 698 (59.7%)	3, 893 (59.1%)	1, 984 (42.7%)	1, 779 (51.3%)	1, 797 (60.7%)	1, 546 (43.4%)	546 (52.6%)
Race															
Asian	538 (5.5%)	294 (3.7%)	558 (8.3%)	298 (3.7%)	226 (2.5%)	256 (3.9%)	319 (3.7%)	370 (2.7%)	107 (3.8%)	475 (7.2%)	181 (3.9%)	298 (8.6%)	221 (7.5%)	175 (4.9%)	86 (8.3%)
Black	2, 875 (29.6%)	3, 226 (40.3%)	1, 740 (25.8%)	1, 616 (19.9%)	2, 032 (22.1%)	1, 476 (22.5%)	307 (3.5%)	529 (3.9%)	116 (4.1%)	1,195 (18.2%)	984 (21.2%)	756 (21.8%)	706 (23.9%)	930 (26.1%)	211 (20.3%)
Others	1, 013 (10.4%)	651 (8.1%)	714 (10.6%)	1, 137 (14.0%)	1, 286 (14.0%)	1, 127 (17.2%)	189 (2.2%)	329 (2.4%)	90 (3.2%)	873 (13.3%)	596 (12.8%)	535 (15.4%)	335 (11.3%)	395 (11.1%)	133 (12.8%)
White	5, 287 (54.4%)	3, 831 (47.9%)	3, 729 (55.3%)	5, 053 (62.4%)	5, 644 (61.4%)	3, 691 (56.4%)	7, 900 (90.6%)	12, 290 (90.9%)	2, 532 (89.0%)	4,041 (61.4%)	2, 890 (62.1%)	1, 879 (54.2%)	1, 698 (57.4%)	2, 062 (57.9%)	609 (58.6%)
Ethnicity															
Hispanic or Latino	518 (5.3%)	396 (4.9%)	395 (5.9%)	1, 485 (18.3%)	1, 737 (18.9%)	1, 397 (21.3%)	374 (4.3%)	742 (5.5%)	168 (5.9%)	2,447 (37.2%)	1, 607 (34.6%)	1, 030 (29.7%)	632 (21.4%)	743 (20.9%)	236 (22.7%)
Not Hispanic or Latino	9, 195 (94.7%)	7, 606 (95.1%)	6, 346 (94.1%)	6, 619 (81.7%)	7, 451 (81.1%)	5, 153 (78.7%)	8, 341 (95.7%)	12, 776 (94.5%)	2, 677 (94.1%)	4,137 (62.8%)	3, 044 (65.4%)	2, 438 (70.3%)	2, 328 (78.6%)	2, 819 (79.1%)	803 (77.3%)
Entry Year															
2019	1, 066 (11.0%)	1, 760 (22.0%)	2, 176 (32.3%)	1, 318 (16.3%)	1, 201 (13.1%)	2, 164 (33.0%)	766 (8.8%)	1, 391 (10.3%)	1, 063 (37.4%)	865 (13.1%)	1, 052 (22.6%)	1, 084 (31.3%)	263 (8.9%)	383 (10.8%)	253 (24.4%)
2020	1, 024 (10.5%)	1, 121 (14.0%)	1, 285 (19.1%)	1, 031 (12.7%)	917 (10.0%)	1, 287 (19.6%)	1,042 (12.0%)	1, 572 (11.6%)	609 (21.4%)	787 (12.0%)	818 (17.6%)	766 (22.1%)	291 (9.8%)	324 (9.1%)	195 (18.8%)
2021	1, 053 (10.8%)	791 (9.9%)	778 (11.5%)	1, 313 (16.2%)	1, 409 (15.3%)	1, 046 (16.0%)	1,544 (17.7%)	2, 229 (16.5%)	531 (18.7%)	1,110 (16.9%)	753 (16.2%)	606 (17.5%)	442 (14.9%)	444 (12.5%)	179 (17.2%)
2022	1, 868 (19.2%)	1, 317 (16.5%)	1, 154 (17.1%)	1, 426 (17.6%)	1, 536 (16.7%)	897 (13.7%)	1,844 (21.2%)	2, 513 (18.6%)	301 (10.6%)	1,403 (21.3%)	847 (18.2%)	483 (13.9%)	540 (18.2%)	593 (16.6%)	169 (16.3%)
2023	2, 321 (23.9%)	1, 898 (23.7%)	760 (11.3%)	1, 556 (19.2%)	2, 089 (22.7%)	648 (9.9%)	2, 065 (23.7%)	3, 299 (24.4%)	228 (8.0%)	1,730 (26.3%)	918 (19.7%)	398 (11.5%)	654 (22.1%)	978 (27.5%)	978 (27.5%)
2024	2, 381 (24.5%)	1, 115 (13.9%)	588 (8.7%)	1, 460 (18.0%)	2, 036 (22.2%)	508 (7.8%)	1,454 (17.7%)	2, 514 (18.6%)	113 (4.0%)	689 (10.5%)	263 (5.7%)	131 (3.8%)	770 (26.0%)	840 (23.6%)	106 (10.2%)
Number of Inpatient Health Utilization															
0	8, 206 (84.5%)	7, 280 (91.0%)	5, 619 (83.4%)	6, 423 (79.3%)	8, 365 (91.0%)	5, 204 (79.5%)	6, 978 (80.1%)	11, 303 (83.6%)	2,418 (85.0%)	6,584 (100.0%)	4, 651 (100.0%)	3, 468 (100.0%)	2, 619 (88.5%)	3, 304 (92.8%)	938 (90.3%)
1	89 (0.9%)	63 (0.8%)	103 (1.5%)	1, 354 (16.7%)	693 (7.5%)	1, 105 (16.9%)	1, 293 (14.8%)	1, 661 (12.3%)	305 (10.7%)	< 10	< 10	< 10	264 (8.9%)	181 (5.1%)	81 (7.8%)
2	178 (1.8%)	60 (0.7%)	140 (2.1%)	233 (2.9%)	101 (1.1%)	169 (2.6%)	313 (3.6%)	417 (3.1%)	95 (3.3%)	< 10	< 10	< 10	51 (1.7%)	52 (1.5%)	11 (1.1%)
> 2	1240 (12.8%)	599 (7.5%)	879 (13.0%)	94 (1.2%)	29 (0.3%)	72 (1.1%)	131 (1.5%)	137 (1.0%)	27 (0.9%)	< 10	< 10	< 10	26 (0.9%)	25 (0.7%)	< 10
Number of Outpatient Health Utilization															
0	42 (0.4%)	20 (0.2%)	40 (0.6%)	1, 111 (13.7%)	730 (7.9%)	1, 238 (18.9%)	< 10	< 10	< 10	6, 584 (100.0%)	4, 651 (100.0%)	3, 468 (100.0%)	47 (1.6%)	28 (0.8%)	< 10
1	557 (5.7%)	386 (4.8%)	502 (7.4%)	1609 (19.9%)	1, 837 (20.0%)	1, 388 (21.2%)	81 (0.9%)	94 (0.7%)	32 (1.1%)	< 10	< 10	< 10	775 (26.2%)	918 (25.8%)	327 (31.5%)
2	462 (4.8%)	277 (3.5%)	385 (5.7%)	1, 296 (16.0%)	1, 539 (16.8%)	1, 083 (16.5%)	69 (0.8%)	106 (0.8%)	37 (1.3%)	< 10	< 10	< 10	478 (16.1%)	620 (17.4%)	164 (15.8%)
> 2	8, 652 (89.1%)	7, 319 (91.5%)	5, 814 (86.2%)	4, 088 (50.4%)	5, 082 (55.3%)	2, 841 (43.4%)	8, 560 (98.2%)	13, 313 (98.5%)	2, 774 (97.5%)	< 10	< 10	< 10	1660 (56.1%)	1996 (56.0%)	541 (52.1%)
Number of ED Health Utilization															
0	8, 874 (91.4%)	7, 223 (90.3%)	6, 183 (91.7%)	6, 087 (75.1%)	6, 793 (73.9%)	4, 660 (71.1%)	2, 308 (81.1%)	10, 674 (79.0%)	2, 308 (81.1%)	6, 584 (100.0%)	4, 651 (100.0%)	3, 468 (100.0%)	2, 806 (94.8%)	3, 362 (94.4%)	985 (94.8%)
1	163 (1.7%)	167 (2.1%)	119 (1.8%)	1, 416 (17.5%)	1, 681 (18.3%)	1, 299 (19.8%)	64 (2.2%)	286 (2.1%)	64 (2.2%)	< 10	< 10	< 10	124 (4.2%)	163 (4.6%)	42 (4.0%)
2	257 (2.6%)	235 (2.9%)	165 (2.4%)	379 (4.7%)	475 (5.2%)	360 (5.5%)	266 (9.3%)	1, 562 (11.6%)	266 (9.3%)	< 10	< 10	< 10	27 (0.9%)	29 (0.8%)	< 10
> 2	419 (4.3%)	377 (4.7%)	274 (4.1%)	222 (2.7%)	239 (2.6%)	231 (3.5%)	207 (7.3%)	996 (7.4%)	207 (7.3%)	< 10	< 10	< 10	< 10	< 10	< 10
Vital Signs															
Hemoglobin A1C (HbA1c)	8.14 (1.48)	8.19 (1.76)	8.21 (1.43)	8.08 (1.71)	7.85 (1.81)	8.35 (1.78)	8.09 (1.35)	8.14 (1.50)	8.34 (1.30)	8.25 (1.71)	8.28 (1.70)	8.31 (1.63)	7.77 (1.34)	7.82 (1.51)	7.87 (1.23)
Body Mass Index (BMI)	32.37 (6.55)	36.20 (7.12)	31.42 (6.31)	32.42 (6.48)	37.19 (7.40)	31.88 (6.54)	32.47 (8.45)	35.67 (9.35)	31.62 (9.35)	31.71 (6.21)	35.04 (6.27)	31.13 (6.18)	33.17 (4.42)	35.07 (5.00)	32.49 (4.37)
Systolic Blood Pressure (SBP)	130.73 (16.30)	131.52 (15.68)	132.19 (16.30)	131.14 (16.61)	131.26 (15.31)	132.91 (16.97)	131.87 (16.09)	132.10 (14.84)	131.88 (15.41)	130.40 (15.86)	131.59 (13.30)	132.62 (15.47)	132.09 (9.91)	131.15 (10.31)	133.50 (10.32)
Diastolic Blood Pressure (DBP)	76.80 (9.72)	78.95 (9.49)	76.30 (9.74)	76.41 (9.97)	78.31 (9.30)	76.38 (9.93)	76.00 (9.69)	78.02 (9.14)	75.77 (9.62)	73.91 (9.90)	76.16 (8.96)	73.59 (10.15)	77.65 (5.90)	79.24 (5.87)	77.61 (5.82)
Medication Use															
Insulin	2, 896 (29.8%)	2, 310 (28.9%)	2, 144 (31.8%)	2, 749 (33.9%)	2, 638 (28.7%)	2, 344 (35.8%)	< 10	< 10	< 10	1,899 (28.8%)	1, 402 (30.1%)	946 (27.3%)	848 (28.6%)	789 (22.2%)	275 (26.5%)
Metformin	5, 585 (57.5%)	4, 802 (60.0%)	4, 294 (63.7%)	4, 611 (56.9%)	4, 830 (52.6%)	4, 405 (67.3%)	< 10	< 10	< 10	3,611 (54.8%)	2, 622 (56.4%)	2, 166 (62.5%)	1, 144 (38.6%)	1, 405 (39.4%)	449 (43.2%)
Lipid	7, 038 (72.5%)	5, 041 (63.0%)	4, 713 (69.9%)	5, 509 (68.0%)	4, 945 (53.8%)	4, 043 (61.7%)	< 10	< 10	< 10	4, 298 (65.3%)	2, 445 (52.6%)	2, 048 (59.1%)	1, 626 (54.9%)	1, 638 (46.0%)	574 (55.2%)
Opioids	2, 330 (24.0%)	1, 631 (20.4%)	1, 689 (25.1%)	1, 314 (16.2%)	1,930 (21.0%)	1, 151 (17.6%)	< 10	< 10	< 10	1,353 (20.5%)	811 (17.4%)	740 (21.3%)	874 (29.5%)	884 (24.8%)	337 (32.4%)
Medical History															
Chronic Kidney Disease	1,904 (19.6%)	763 (9.5%)	1, 123 (16.7%)	2, 126 (26.2%)	759 (8.3%)	1,048 (16.0%)	< 10	< 10	< 10	1,120 (17.0%)	426 (9.2%)	562 (16.2%)	< 10	< 10	< 10
Hypertension	7, 333 (75.5%)	5, 544 (69.3%)	4, 984 (73.9%)	4, 816 (59.4%)	1, 201 (13.1%)	1, 235 (18.9%)	< 10	< 10	< 10	5,007 (76.0%)	3, 155 (67.8%)	2, 630 (75.8%)	< 10	< 10	< 10
Obesity Disorder	2, 691 (27.7%)	3, 813 (47.7%)	1, 454 (21.6%)	3, 364 (41.5%)	403 (4.4%)	512 (7.8%)	< 10	< 10	< 10	2,731 (41.5%)	2, 813 (60.5%)	1, 214 (35.0%)	< 10	< 10	< 10
Sleep Disorder	1, 904 (19.6%)	1, 849 (23.1%)	935 (13.9%)	4, 198 (51.8%)	6, 382 (69.5%)	5, 028 (76.8%)	2,619 (30.1%)	4, 569 (33.8%)	688 (24.2%)	1,352 (20.5%)	1, 109 (23.8%)	556 (16.0%)	711 (24.0%)	1,069 (30.0%)	195 (18.1%)

## Data Availability

The data that support the findings of this study are not publicly available due to data use agreements with participating health systems but are available from the corresponding author upon reasonable request.
